# Renin–angiotensin system gene polymorphisms among Saudi patients with coronary artery disease

**DOI:** 10.1186/2241-5793-21-8

**Published:** 2014-05-21

**Authors:** Amal Al-Hazzani, Mohamed S Daoud, Farid S Ataya, Dalia Fouad, Abdulaziz A Al-Jafari

**Affiliations:** Department of Botany and Microbiology, College of Science, King Saud University, P.O. Box 22452, Riyadh, 11459 Saudi Arabia; Department of Biochemistry, College of Science, King Saud University, P.O. Box 2455, Riyadh, 11451 Saudi Arabia; King Fahd Unit Laboratory, Department of Clinical and Chemical Pathology, Kasr Al-Ainy University Hospital, Cairo University, El-Manial, Cairo, 11562 Egypt; Department of Molecular Biology, Genetic Engineering Division, National Research Center, Dokki, Cairo, 12311 Egypt; Department of Zoology, College of Science, King Saud University, Riyadh, Saudi Arabia; Department of Zoology and Entomology, Faculty of Science, Helwan University, Ein Helwan, Cairo, Egypt

**Keywords:** Coronary artery disease, Angiotensin, Genotypes, Angiotensin converting enzyme, Angiotensin receptors, Saudi populations and polymorphism

## Abstract

**Background:**

The polymorphisms in the components of the renin-angiotensin system (RAS) are important in the development and progression of coronary artery disease (CAD) in some individuals. Our objectives in the present investigation were to determine whether three RAS polymorphisms, angiotensin-converting enzyme insertion/deletion (ACE I/D), angiotensin receptor II (Ang II AT2 - C3123A) and angiotensinogen (AGT-M235T), are associated with CAD in the Saudi population. We recruited 225 subjects with angiographically confirmed CAD who had identical ethnic backgrounds and 110 control subjects. The polymerase chain reaction-restriction fragment length polymorphisms (RFLP) technique was used to detect polymorphisms in the RAS gene.

**Results:**

Within the CAD group, for the ACE I/D genotype, DD was found in 64.4%, 26.3% carried the ID genotype, and 9.3% carried the II genotype. Within the control group, the DD genotype was found in 56.4%, 23.6% carried the ID genotype, and 20% carried the II genotype. The odds ratio (OR) of the ACE DD *vs* II genotype with a 95% confidence interval (CI) was 2.45 (1.26-4.78), with *p* = 0.008. For the Ang II AT2 receptor C3123A genotype, within the CAD group, CC was found in 39.6%, 17.8% carried the CA genotype, and 42.6% carried the AA genotype. Within the control group, CC was found in 39.1%, 60.9% carried the CA genotype, and there was an absence of the AA genotype. The OR of the Ang II AT2 receptor C3123A CC *vs* AA genotypes (95% CI) was 0.01, with *p* = 0.0001. A significant association with CAD was shown. For the AGT-M235T genotype, within the CAD group, MM was found in 24.0%, 43.6% carried the MT genotype and 32.4% carried the TT genotype. Within the control group, MM was found in 26.4%, 45.5% carried the TT genotype and 28.2% carried the MT genotype. The OR of MM *vs* TT (95% CI) was 0.79 (0.43 to 1.46), which was insignificant.

**Conclusions:**

There is an association between the ACE I/D and Ang II AT2 receptor C3123A polymorphisms and CAD, however, no association was detected between the AGT M235T polymorphism and CAD in the Saudi population.

## Background

The renin-angiotensin system, which regulates blood pressure, plays a pivotal role in the pathogenesis of CAD [1]. Several studies have suggested that polymorphisms in the components of the renin-angiotensin system (RAS) are important in the development and progression of CAD in some individuals. This has been supported by the evidence of the efficacy of angiotensin-converting enzyme (ACE) inhibitors and angiotensin-II receptor blockers (ARBs) in halting the development of coronary atherosclerosis and related coronary events [2]. ACE, a key component of the RAS, is a peptidase that cleaves the histidyl-leucine dipeptide from inactive angiotensin I. It is well documented that angiotensin-converting enzyme (ACE, EC 3.4.15.1) gene polymorphisms are associated with various diseases such as hypertension, coronary artery disease, myocardial infarction and diabetes [3].

Angiotensin I (Ang I) generates vasoactive angiotensin II, which is a potent vasopressor. Angiotensin II affects the contractility and growth of the vascular endothelium and vascular smooth muscle cells (VSMC) and plays a role in the coronary atherosclerotic process in the development of the hyperplastic and hypertrophic VSMC proliferation and migration; this results in stimulation of the synthesis of plasminogen activator inhibitor-1 by fibroblasts, which results in chronic and acute coronary disorders [4]. Several studies have suggested that the major components of the RAS, ACE and Ang II, possess considerable effects in cardiovascular disease processes and might be modulated by some components of gene abnormalities and disorders. This is supported in part by the results of association studies that focused on the involvement of polymorphisms in the genes of the RAS pathway components and cardiac disease disorders [5].

Various studies have reported a relationship between ACE gene I/D polymorphisms and cardiovascular disorders. A report by Cambien et al. in 1992 first predicted the strong relationship of the ACE D allele as an independent risk factor for myocardial infarction (MI) [3], and studies were later conducted intensively to investigate the relationship between ACE gene I/D polymorphisms and CAD in different individuals from different populations, yet their results were inconsistent [6, 7]. These variations are likely due to various environmental and genetic factors that have not been explored or investigated separately. However, the relationship between polymorphisms in Ang II, AT1, and AT2 receptors and CAD has been reported by several investigators [8, 9]. An angiotensinogen (AGT) gene polymorphism (M235T) has been proposed to be associated with CAD [10, 11]. Given this background, the aim of the present study was to assess the possible association between, angiotensin-converting enzyme insertion/deletion (ACE I/D), angiotensin receptor II (Ang II AT2-C3123A) and angiotensinogen (AGT-M235T) in Saudi patients with coronary artery disease as confirmed by coronary angiography diagnosis, because the contribution of these RAS polymorphisms to the pathogenesis of CAD has not been studied previously in Saudi CAD patients.

## Results

### Demographic characteristics of the control subjects and the CAD patients

Two hundred twenty-five CAD patients and one hundred and ten control subjects were studied. Table 1 shows their clinical characteristics. There was a significant difference between the CAD patients and the control subjects with regard to age, gender, plasma fasting blood sugar (FBS), triglycerides (TG), low-density lipoprotein-cholesterol (LDL-c) (*p* < 0.0001) and TC (*p* < 0.001). There was no difference between the CAD patients and the control subjects in the high-density lipoprotein-cholesterol (HDL-c) (*p* =0.34).

**Table 1:** **Demographic characteristics of the control subjects and the CAD patients**

Characteristic	Controls	CAD group	***p*** level
	n = 110	n = 225	
**Age, years**			
**Mean ± SD**	46.61 ± 16.15	61.22 ± 10.35	< 0.0001
**Range**	(20.0-78.0)	(31.0-89.0)	
**Gender**			
**Male (%)**	62 (56.4%)	153 (68.0%)	< 0.0001
**Female (%)**	48 (43.6%)	72 (32.0%)	
**FBS, mmol l** ^**−1**^			
**Mean ± SD**	4.48 ± 0.64	8.08 ± 3.41	< 0.0001
**Range**	(3.20-7.10)	(3.3-20.6)	
**TG, mmol l** ^**−1**^			
**Mean ± SD**	1.11 ± 0.27	1.87 ± 1.19	< 0.0001
**Range**	(0.50-1.90)	(0.60-8.70)	
**TC, mmol l** ^**−1**^			
**Mean ± SD**	3.81 ± 0.54	4.16 ± 0.99	< 0.001
**Range**	(3.00-7.10)	(0.80-7.5)	
**HDL-c, mmol l** ^**−1**^			
**Mean ± SD**	1.24 ± 0.36	1.15 ± 0.95	0.340
**Range**	(0.80-2.20)	(0.50-10.7)	
**LDL-c, mmol l** ^**−1**^			
**Mean ± SD**	1.65 ± 0.59	2.37 ± 0.85	< 0.0001
**Range**	(0.90-4.50)	(0.60-5.90)	

The Student’s *t*-test and the *χ* ^2^ test were used to compare the values of the controls and the CAD patients.

### CAD risk factors in the patients and the control subjects

Other demographic characteristics are listed in Table 2. There were significant differences between the CAD patients and the control group with regard to diabetes mellitus, dyslipidemia, hypertension, and smoking. Using the *χ*^*2*^ test, diabetes mellitus (*p* < 0.0001, OR = 20.34, 95% CI: 9.78-4.24), dyslipidemia (*p* < 0.0001, OR = 9.38, 95% CI: 5.05-17.44), hypertension (*p* < 0.0001, OR = 22.46, 95% CI: 11.51-43.81), and smoking (*p* < 0.0001, OR = 3.85, 95% CI: 2.12-6.96) were found to be independent risk factors of CAD.

**Table 2:** **CAD risk factors in the patients and the control subjects**

Parameter	CAD	Control	OR	95% CI	***p*** level
	(n = 225)	(n = 110)			
**Diabetes mellitus**					
Diabetic	145 (64.4%)	9 (8.2%)			
Non-diabetic	80 (35.6%)	101(91.8%)	20.34	9.78-42.40	< 0.0001
**Dyslipidemia**					
Positive	130 (57.8%)	14 (12.7%)			
Negative	95 (42.2%)	96 (87.3%)	9.38	5.05-17.44	< 0.0001
**Hypertension**					
Hypertensive	165 (73.3%)	12 (10.9%)			
Normotensive	60 (26.7%)	98 (89.1%)	22.46	11.51-43.81	< 0.0001
**Smoking**					
Smoker	89 (39.6%)	16 (14.5%)			
Non-smoker	136 (60.4%)	94 (85.5%)	3.85	2.12-6.96	< 0.0001

### ACE I/D, Ang II AT2 receptor C3123A and AGT M235T genotype distributions and allele frequencies in Saudi CAD and healthy patients

Genotype frequencies did not deviate from Hardy-Weinberg expectations in both controls and CAD group. The genotype frequencies are listed in Table 3. A significant difference in the genotype distribution of ACE I/D and Ang II AT2 receptor C3123A polymorphisms were observed between the CAD patients the and control subjects (*p* = 0.023 and 0.0001, respectively), however no significant differences were observed in the genotype distribution of AGT M235T between the CAD patients and the control subjects (*p* = 0.102). Table 4 shows the significant differences in D and I and in C and A allele distributions observed between the CAD and the control groups (*p* = 0.009 and 0.0001, respectively). No significant differences in M and T allele distributions were observed between the CAD and the control groups (*p* = 0.419).

**Table 3:** **ACE I/D, Ang II AT2 receptor C3123A, and AGT M235T genotype distributions in CAD and healthy patients**

Genotype	Groups			***p*** value
	Control	CAD patients	Total	
	(n = 110)	(n = 225)	(n = 335)	
**ACE I/D**				
**DD**	62 (56.4%)	145 (64.4%)	207 (61.79%)	0.023
**ID**	26 (23.6%)	59 (26.3%)	85 (25.37%)	
**II**	22 (20.0%)	21 (9.3%)	43(12.84%)	
**Ang II AT2** **(C3123A)**				
**CC**	43 (39.1%)	89 (39.6%)	132 (39.40%)	0.0001
**CA**	67 (60.9%)	40 (17.8%)	107 (31.94%)	
**AA**	0	96 (42.6%)	96 (28.66%)	
**AGT (M235T)**				
**MM**	29 (26.40%)	54 (24.0%)	83 (24.78%)	0.102
**MT**	50 (45.50%)	98 (43.6%)	148 (44.18%)	
**TT**	31 (28.20%)	73 (32.4%)	104 (31.04%)	

The *χ* ^2^ test was used to compare the genotype distributions between the control and CAD patients.

**Table 4:** **ACE I/D, Ang II AT2 receptor C3123A, and AGT M235T allele frequencies in CAD and healthy patients**

Alleles	Groups		Total	***p*** value
	Control	CAD patients		
	(n = 110)	(n = 225)		
**ACEI/D**				
**D**	150 (68.18%)	349 (77.56%)	499 (74.48%)	0.009
**I**	70 (31.82%)	101 (22.44%)	171 (25.52%)	
***Total***	220	450	670	
**Ang II AT2** (**C3123A)**				
**C**	153 (69.54%)	218 (48.44%)	371 (55.37%)	0.0001
**A**	67 (30.46%)	232 (51.56%)	299 (44.63%)	
***Total***	220	450	670	
**AGT (M235T)**				
**M**	108 (49.09%)	206 (45.78%)	314 (46.87%)	0.419
**T**	112 (50.91%)	244 (54.22%)	356 (53.13%)	
***Total***	220	450	670	

The *χ* ^2^ test was used to compare the allele frequencies between the control and CAD patients.

### CAD odds ratio associations with ACE I/D, Ang II AT2 receptor C3123A and AGT M235T genotypes

The odds ratios of the ACE I/D genotype DD *vs* II, DD + ID *vs* II and ID *vs* II genotypes (95% CI) were 2.45 (1.26-4.78), 2.43 (1.27-4.64), and 2.38 (1.12-5.06). These results demonstrate a significant association with CAD disease (*p* = 0.008, 0.007 and 0.02, respectively). The odds ratios of the Ang II AT2 receptor C3123A genotype CC *vs* CA and CC + AA *vs* CA (95% CI) were 3.45 (2.03-5.92) and 7.21 (4.31-12.04), respectively, which shows a significant association with CAD disease (p < 0.0001). The odds ratio of the ACT M235T genotype MM *vs* MT, MM *vs* TT, MM *vs* MT + TT, and MM + TT *vs* MT (95% CI) were 0.95 (0.54-1.67), 0.79 (0.43-1.46), 0.88 (0.52-1.49), and 1.08 (0.68-1.71), respectively, indicating that there was no significant association with CAD disease (Table 5).

**Table 5:** **CAD odds ratio associations with ACE I/D, Ang II AT2 receptor C3123A, and AGT M235T genotypes**

	OR	95% CI	***p value***
**ACE I/D genotypes**			
ID *vs* II	2.38	(1.12-5.06)	0.02
DD *vs* II	2.45	(1.26-4.78)	0.008
DD *vs* ID	1.03	(0.60-1.78)	0.914
DD *vs* ID and II	1.40	(0.88-2.23)	0.15
DD and ID *vs* II	2.43	(1.27-4.64)	0.007
**Ang II AT2 (C3123A) genotypes**			
CC *vs* CA	3.45	(2.03-5.92)	< 0.0001
CC *vs* AA	0.01	(0.001-0.18)	0.001
CC *vs* CA and AA	1.02	(0.64-1.63)	0.93
CC and AA *vs* CA	7.21	(4.31-12.04)	< 0.0001
**AGT (M235T) genotypes**			
MM *vs* MT	0.95	(0.54-1.67)	0.86
MM *vs* TT	0.79	(0.43-1.46)	0.46
TT *vs* MT	1.20	(0.70-2.06)	0.51
MM *vs* MT and TT	0.88	(0.52-1.49)	0.64
MM and TT *vs* MT	1.08	(0.68-1.71)	0.74

CI = confidence interval.

### Frequencies of the ACE I/D, Ang II AT2 receptor C3123A, and ACT M235T genotype combinations in the CAD and control groups

Our study revealed 26 ACE I/D, Ang II AT2 receptor (C3123A), and ACT (M235T) genotype combinations. The DDAAMT (OR = 30.62, 95% CI = 1.85-506.8, *p* = 0.016), DDAAMM (OR = 23.23, 95% CI = 1.39-387.2, *p* = 0.028), DDAATT (OR = 19.70, 95% CI = 1.18-330.1, *p* = 0.038), IDAAMT, IDCCTT, IDAATT, IDAAMT, IIAAMT, and IIAATT genotype combinations were observed only in the CAD group. The DDACTT, IDACMT, IDACMM, DDACMM, IICCTT, and IIACTT genotype combinations were significantly more common in the control group compared with the CAD group (*p* = 0.018, 0.016, 0.038, 0.005, 0.019 and 0.023, respectively) (Table 6).

**Table 6:** **Genotype combination frequencies of the ACE I/D, Ang II AT2 receptor C3123A, and AGT M235T**

Genotype combination	CAD (n = 225)	Controls (n = 110)	OR	95% CI	***p***
	n (%)	n (%)			
DDAAMT	27 (12.0%)	0	30.62	1.85-506.8	0.016
DDCCTT	23 (10.22%)	15 (13.64%)	0.72	0.36-1.44	0.356
DDAAMM	21 (9.33%)	0	23.23	1.39-387.2	0.028
DDCCMT	20 (8.89%)	7 (6.36%)	1.44	0.59-3.50	0.427
DDAATT	18 (8.0%)	0	19.70	1.18-330.1	0.038
DDACMT	13 (5.78%)	13 (11.82%)	0.46	0.204-1.02	0.057
DDCCMM	11 (4.89%)	6 (5.45%)	0.89	0.32-2.48	0.825
IDCCMT	11 (4.89%)	1 (0.91%)	5.60	0.71-43.96	0.101
IDAAMT	10 (4.44%)	0	10.77	0.63-185.5	0.101
DDACTT	9 (4.0%)	12 (10.91%)	0.34	0.14-0.83	0.018
IDCCTT	7 (3.11%)	0	7.59	0.43-134.0	0.167
IDAATT	7 (3.11%)	0	7.59	0.43-134.0	0.167
IICCMT	6 (2.67%)	3 (2.73%)	0.98	0.24-3.98	0.974
IDAAMT	6 (2.67%)	0	6.54	0.37-117.2	0.201
IDACTT	5 (2.22%)	7 (6.36%)	0.33	0.10-1.08	0.067
IICCMM	5 (2.22%)	3 (2.73%)	0.81	0.19-3.46	0.776
IDCCMM	5 (2.22%)	2 (1.82%)	1.23	0.23-6.43	0.808
IDACMT	5 (2.22%)	9 (8.18%)	0.26	0.08-0.78	0.016
IIAAMT	4 (1.78%)	0	4.49	0.24-84.14	0.315
IDACMM	4 (1.78%)	7 (6.36%)	0.27	0.08-0.93	0.038
DDACMM	3 (1.33%)	9 (8.18%)	0.15	0.04-0.57	0.005
IIAATT	3 (1.33%)	0	3.51	0.18-68.52	0.408
IIACMT	1 (0.44%)	2 (1.82%)	0.24	0.02-2.68	0.248
IICCTT	1 (0.44%)	6 (5.45%)	0.08	0.01-0.65	0.019
IIACMM	0	2 (1.82%)	0.10	0.001-2.02	0.132
IIACTT	0	6 (5.45%)	0.04	0.002-0.64	0.023

## Discussion

The renin-angiotensin system (RAS) has a prominent role in the physiological functions of cardiovascular system and in the pathophysiology of heart diseases such as CAD [12]. CAD is a polygenic disease, the onset and severity of CAD depends on the interaction of many genetic and environmental factors [13]. The association of these RAS gene polymorphisms with classical risk factors including hypertension, obesity, diabetes, and hyperlipidemia has been reported [14–18]. In this study, diabetes mellitus, dyslipidemia, hypertension, and smoking were found to be risk factors for CAD (odds 20.34, 9.38, 22.46 and 3.85, respectively, *p* < 0.0001). Previous studies had indicated an association of the DD genotype with CAD in high-risk patients diagnosed with diabetes mellitus [19]. The DD genotype (*vs* the II genotype) independently increased the risk of CAD in diabetes 2.1-fold, while the ID genotype did not alter the risk significantly [20]. Hyperlipidemia as a major risk factor of CAD increases the plasma concentration of angiotensinogen and the angiotensin peptides II and III and up-regulates the expression of the angiotensin II type 1 receptor (AT1R) gene [16]. Although the positive relationship between the DD genotype, the D allele frequency and hyperlipidemia was demonstrated by prior studies [17, 21], Oren *et al*. reported higher LDL cholesterol levels in patients with the DD genotype, intermediate levels in the ID patients, and lower levels in the II patients [18]. Other studies did not find any correlation between the lipid profile and polymorphisms [17, 19, 22, 23]. The ACE I/D polymorphism has been extensively studied and points to an association with arterial hypertension [24]. Cigarette smoking is another risk factor for CAD, and is particularly common in Turkish patients [14]. Previous data have suggested that nicotine increases ACE expression [25] and the D allele smokers have been found to be associated with endothelial dysfunction [26]. Moreover, smoking patients with ID genotype were found to have an increased risk of CAD and an association between the ID genotype, hyperlipidemia and cigarette smoking has been proposed [13].

Genetic factors play a role in the development of CAD but differ among various populations. The ACE I/D gene polymorphisms are the most frequently studied and have been proposed as CAD risk factors [27]. In the present study, samples from CAD patients and controls were investigated to assess the relationship between three RAS polymorphisms with CAD in a sample of Saudi patients. We found that the ACE D and I alleles differ significantly between CAD group and controls (*p* = 0.009) and a significant association between the DD genotype polymorphisms and CAD (*p* = 0.008, OR: 2.45, 95% CI = 1.26-4.78) was observed. Our results are consistent with previous studies (although the relationship between CAD and ACE gene polymorphism (DD genotype) was first reported by Cambien *et al*. [3]). Beohar *et al*. considered that the ACE D allele and DD genotype were the major risk factors for CAD [28]. Since then, many studies have found the D allele or DD genotype to be associated with myocardial infarction, coronary heart disease [8] or other cardiovascular pathology, hypertrophic cardiomyopathy [29], and coronary artery stenosis [30]. The DD polymorphism was more closely associated with CAD than the other two genotypes (ID and II) in CAD patients [31]. On the other hand, several studies found no association with the occurrence of either CAD or MI [32]. There were relationships between the presence of CAD and the ACE D allele in a large case-controlled study [33]. Ethnic differences can explain the disparity between prior clinical studies. In 2005, Acartürk *et al*. showed that the DD genotype is a significant predictor of CAD in a population living in Southern Turkey [14]. The DD genotype of the ACE I/D gene has been reported as a risk factor for the development of various heart diseases in Caucasian, Chinese, and Australian populations [34, 35]. The DD genotype frequency of the ACE I/D polymorphism was markedly higher in CAD depressed Iranian patients than in the non-CAD depressed control group, and it was associated with a 2.32-fold increased risk of CAD. The DD genotype of ACE I/D (*vs* the II genotype) could independently and strongly increase the risk of CAD by 9.4-fold in depressed CAD individuals [11]. The ACE I/D polymorphism did not play a role in the development of CAD or MI in a Western, Australian and Caucasian population [27, 28]. Some studies indicated the lack of an association between the DD genotype and CAD in low risk populations [36].

AT2 receptor is believed to be increased under some pathological conditions such as hypertension, vascular injury, and stroke [19]. In the present study, CAD was associated with AT2 receptor C3123A genetic polymorphism in accordance with a previous study between an Ang II AT2 receptor polymorphism (C3123A) and CAD [37]. However, other authors have failed to show any associations [38]. Firouzabadi *et al*. showed higher frequency of the AA genotype (C3123A) of AT2 receptors, but no association was observed between these genotypes and CAD among CAD depressed patients [11]. This might be due to the low expression of these variants in most populations studied, and these polymorphisms may become associated with CAD in studies with larger sample sizes [11]. Japanese men carried (A) allele of the C3123A polymorphism which was observed to be associated with an increase in blood pressure whereas carriers of the (C) allele did not show this association [39].

The distribution of the angiotensinogen (AGT) genotype is an ethnic difference. Asians and Blacks have higher frequencies of T235 homozygosity than the Caucasian population [40]. The AGT gene polymorphisms (M235T) have been proposed to be associated with CAD [10] and a meta-analysis that included twelve studies demonstrated no association in this regard [41]. Angiotensinogen-235 T was present in 19% of the control population compared with 15% of the individuals in Western populations, and an association was seen between the AGT gene and the risk for coronary heart disease (CHD) [42]. The presence of the AGT M235 homozygote was associated with a 2-fold increase of myocardial infarction risk. In the Spanish and New Zealand populations, T235 homozygosity was associated with an increased risk of CAD [43, 44]. In the present study, the genotype polymorphism AGT M235T (MM, MT and TT) frequencies in Saudi CAD patients were 24, 43.6 and 32.4%, respectively and there was no significant difference between the M and T alleles and no significant association with CAD disease was observed. Kuo *et al*. found that the AGT M235T polymorphism was not related to the presence of CAD. In the same study the AGT genotypes were MM in 3.7%, MT in 49.5%, and TT in 46.7% in the control group, which are comparable to our investigation [45]. However, the presence of T235 homozygosity of the AGT gene was not associated with the existence of CAD but was associated with an increased risk of CHD and essential hypertension in a Japanese population [39, 45]. In contrast, it was associated with CAD in white Europeans [44].

The combined set of RAS alleles ACE I/D D/AGT235 T/AT1R A was the only parameter which was found to be significantly increased as a risk factor of CAD in the whole population analysis studied before [15]. The interaction between AGT TT and ACE ID genotypes has been previously observed among no diabetic patients with clinically diagnosed CAD [41]. Sekuri *et al*. demonstrated that an increased premature CHD risk is associated with higher frequencies of the ACE DD and AGT MM genotypes [46]. In our study, the genotype combinations, DDAAMT, DDAAMM and DDAATT were observed only in the CAD group compared to the wild type. Also it is well documented that the RAS genetic polymorphisms (ACE DD, AGT TT, and ATR1 CC) may increase the susceptibility of an individual to have premature CAD [38].

## Conclusions

We found an association between the ACEI/D and Ang II AT2 receptor C3123A polymorphisms and CAD, but we did not find an association between the AGT M235T polymorphism and CAD. A combination of genetic and environmental factors may influence the onset of CAD, and RAS gene polymorphisms have a strong role in the development of CAD. Further studies with a larger study population on other RAS gene polymorphisms are necessary for patients with CAD in order to investigate the possible effects.

## Methods

### Study subjects

Two hundred twenty-five CAD patients (156 males and 69 females, aged 42–82 years old) who were admitted to Department of Cardiology, King Khalid University Hospital, Riyadh, Saudi Arabia and a control group of 110 healthy subjects (59 males and 51 females, aged 20–78 years old) who had no history of CAD were included in this study. The included subjects were of unrestricted age and gender and provided written informed consent for drawing blood at the time of angiography or at the time of screening for research deoxyribonucleic acid (DNA) extraction to be used in studies approved by the hospital’s institutional review board. The study was conducted in accordance with the guidelines set by the ethics committee of College of Medicine and Research Centre (CMRC) of King Saud University, Riyadh, Saudi Arabia. All the subjects enrolled in this study were Saudi residents with similar dietary patterns. The key demographic data of the subjects were recorded including the age, gender, and lipid profile. Assessments of CAD were made by the patients’ cardiologists through the reviewing of angiograms.

### Ethical approval

This study was conducted after review and approval of the Institutional Review Board of the Ethics Committee at KKUH (King Khalid University Hospital), and all subjects gave written informed consent prior to participation.

### Sample collection and lipid analysis

The blood samples for glucose and lipid measurements were drawn from the patients and the control subjects after an overnight fast. The plasma glucose concentration was measured by the glucose oxidase method using a Biotrol Kit (BIOTROL, USA) on a Bayer opera analyzer (Bayer Diagnostics - Siemens, Germany). The serum total cholesterol was measured using the Biotrol commercial Kit. The HDL cholesterol was determined with a commercial Randox Kit (Randox Laboratories Ltd., United Kingdom). The LDL cholesterol was calculated by the formula of Friedwald. The triglyceride determination was made by the method of Lipase/Glycerol Kinase UV endpoint on the opera analyzer.

### DNA extraction

Genomic DNA was extracted from the peripheral blood (in tubes containing EDTA as an anticoagulant) using the QIAamp DNA isolation Kit from QIAGEN (Germany).

### Genotyping and polymorphism analysis

Genotyping of ACE I/D, C3123A, and M235T polymorphisms were determined using polymerase chain reaction restriction fragment length polymorphism (PCR-RFLP) from genomic DNA. The primer sets were selected on the basis of previously published information [2, 3, 47]: ACE I/D, forward primer: 5′-CTG GAG ACC ACT CCC ATC CTT TCT-3′ and reverse primer: 5′-GAT GTG GCC ATC ACA TTC GTC AGT T-3′; Ang II AT2 receptor (C3123A), forward primer: 5′-GGA TTC AGA TTT CTC TTT GAA-3′ and reverse primer: 5′-GCA TAG GAG TAT GAT TTA ATC-3′; AGT (M235T), forward primer: 5′-CAG GGT GCT GTC CAC ACT GGA CCC C-3′ and reverse primer: 5′-CCG TTT GTG CAG GGC CTG GCT CTC T-3′. Genomic DNA template 3 μL (150 ng) was added to the PCR reaction mixture containing 12.5 μL of 2× Promega master mixes, 2 μL of each primer and distilled water to a final volume of 25 μL. The PCR conditions were: initial denaturation at 94°C for 2 min followed by 40 cycles of denaturation at 94°C for 15 s, annealing at 50°C for 30 s, and extension at 72°C for 1 min, and a final extension at 72°C for 2 min in a My Cycler (Bio-Rad). Digestion of the C3123A and M235T PCR products was performed by the addition of 1 μL of the appropriate restriction enzyme (*Alu*I and *Pfl*FI: New England Biolabs Inc., UK) to 10 μL of PCR products in 2 μL of a 10× buffer solution (final reaction volume = 20 μL). The mixture was centrifuged for 2 min at 5000 rpm and kept in a water bath at 37°C overnight. The resulting fragments were resolved by electrophoresis (80 V, 60 min) on 3.0% agarose gels and directly visualized under UV light. For ACE I/D the homozygous individuals for the D allele (DD genotype) were identified by the presence of a single 190 bp PCR product. The homozygous for I allele (II genotype) were identified by the presence of a single 490 bp PCR product. The heterozygous individuals (ID genotype) were identified by the presence of both 190 bp and 490 bp PCR products. For Ang II AT2 receptor (C3123A) the homozygous individuals for the C allele (CC genotype) were identified by the presence of a single 321 bp PCR product. The homozygous for A allele (AA genotype) were identified by the presence of both 214 bp and 107 bp PCR product. The heterozygous individuals (CA genotype) were identified by the presence of 321 bp, 214 bp and 107 bp PCR products. For AGT (M235T) the homozygous individuals for the M allele (MM genotype) were identified by the presence of a single 165 bp PCR product. The homozygous for TT allele (TT genotype) were identified by the presence of both 140 bp and 25 bp PCR product. The heterozygous individuals (MT genotype) were identified by the presence of 165 bp, 140 bp and 25 bp PCR products.

### Statistical analysis

The measurement data were summarized by the mean ± standard deviation (SD) and compared with a two-sample *t*-test. The enumeration count data were summarized as the number (%) and compared with a chi-square test (*χ*^2^ test). Two analyses were used to evaluate the allelic and genotypic frequencies that were calculated from the observed genotypic counts and to assess the Hardy-Weinberg equilibrium expectations. The same methodology was applied to the comparisons between the allelic and genotypic frequencies. Associations were determined as odds ratios (ORs) and 95% confidence intervals (CIs). The odds of carrying a specific allele are defined as the frequency of subjects in whom the allele occurs divided by the frequency of subjects in whom the allele does not occur. An odds ratio for the ACE I/D genotype distribution *χ*^2^ analysis was performed. CAD is the odds of allelic carriage in the diseased [CAD] group divided by the odds in the healthy [control] group. The statistical analysis was performed with the Statistical Package for Social Sciences for Windows, version 20.0 (SPSS, Inc, Chicago, IL, USA).
